# Is there any Association Between Human Lymphotropic Virus Type I (HTLV-I) Infection and Systemic Lupus Erythematosus? An Original Research and Literature Review

**Published:** 2013-03

**Authors:** Abbas Shirdel, Kamila Hashemzadeh, Maryam Sahebari, Houshang Rafatpanah, MohammadReza Hatef, Zahra Rezaieyazdi, Zahra Mirfeizi, Reza FaridHosseini

**Affiliations:** 1 Department of Internal Medicine, Ghaem Hospital, School of Medicine, Mashhad University of Medical Sciences, Mashhad, Iran; 2 Rheumatic Diseases Research Centre, School of Medicine, Mashhad University of Medical Sciences, Mashhad, Iran; 3 Asthma and Allergy Research Centre, School of Medicine, Mashhad University of Medical Sciences, Mashhad, Iran

**Keywords:** HTLV-I, Human T Lymphotropic Virus Type 1, Systemic Lupus Erythematosus, SLE

## Abstract

***Objective(s):*** Systemic lupus erythematosus (SLE) is an autoimmune disease with unknown etiology. Some environmental factors can induce SLE in genetically susceptible individuals; for example, sun exposure and some viral infections may emerge the disease manifestations. Human T lymphotropic virus type 1 (HTLV-I) can dysregulate the human immune system, and the role of this virus in the pathogenesis of autoimmune diseases is under investigation. There are conflicting data about the role of HTLV-I in the pathogenesis of several autoimmune diseases such as SLE. In this study, we have focused on the correlation between HTLV-I infection and SLE in the northeast of Iran, an endemic area for the virus.

***Materials and Methods:*** One hundred and thirty women with SLE and 915 healthy controls were screened for HTLV-I by enzyme linked immunosorbent assay (ELISA). Western blot method was used for confirmation of the positive results done by ELISA in the patients and the control group.

***Results:*** Two (1.5%) of the patients and 23 (2.5%) of the healthy controls were HTLV-I seropositive. There was not a statistical difference between patients and controls in the number of HTLV-I seropositive samples (P=0.49).

***Conclusion:*** This cross-sectional case-control study did not find any association between HTLV-I and SLE. With regard to the previous studies, these controversies may stem from differences in ethnic background. Geographical and environmental factors should also be taken into account.

## Introduction

Human T lymphotropic virus type I (HTLV-I) is a C-type retrovirus which is associated with two main types of disease; HTLV-I-associated myelopathy/tropical spastic paraparesis (HAM/TSP) and adult T cell leukemia (ATL) (1-2).

The virus is endemic in southwestern Japan, parts of Africa, and Central and South America (3-4). Mashhad in the northeast of Iran has been known as a new endemic region of HTLV-I and the prevalence of HTLV-I infection is estimated to be 2-3% in the whole population (5).

This virus with several routes of transmission not only induces HAM/TSP in a small proportion of HTLV-I carriers, but also is associated with other autoimmune diseases (6-7). 

Although pathogenesis of HAM/TSP remains unknown; autoimmune imbalance in the efficiency of HTLV-I-specific cytotoxic T lymphocytes (CTL), HLA associations, and HTLV-I antibody titer (8) bear in mind that HTLV-I can dysregulate immune system. For example, HTLV-I has been considered to play an important role in chronic inflammatory arthritis, named HTLV-I-associated arthropathy (HAAP), and several other autoimmune disorders (9-10). In addition, the role of HTLV-I in the pathogenesis of SLE has been discussed extensively (7, 9-14). SLE is a systemic autoimmune disease of unknown etiology, characterized by autoantibody formation and cell immunity disturbance (15-16). Patients may show altered suppressor T-cell to helper T-cell ratios. Abnormalities in T-cell function include T-cell lymphopenia, impaired apoptosis, hyper-reaction to signaling to T- cell receptors, expression of activated antigens, defects in deletion of cells with high affinity for self-antigens, and alteration of responses to cytokines and lymphokines (11, 15-16). 

Taken together, in addition to escape from self-tolerance, molecular mimicry, and inflammatory cytokines (9, 15-18), several environmental factors especially infections are suspicious for the pathogenesis of SLE (19-22). Elevated titers of antibodies to some viruses such as Epstein Barr virus have been reported in SLE (22). The role of HTLV-I infection in the pathogenesis of SLE is controversial (11, 13-14, 23-33).

Transgenic mice carrying retrovirus specific genes, such as HTLV-I Tax, showed autoimmune-like pathology, suggesting that this virus has the potential to induce autoimmune disorders (34-35). Tax is a nuclear protein encoded by the pX region of HTLV-I and the aberrant expression of cellular genes by Tax is proposed to be essential for the transformation of T cells (9). High titers of HTLV-I antibody can stimulate immune responses which lead to inflammatory tissue damage (8, 36). This hypothesis suggests that inefficient cytotoxic T cell response to HTLV-I leads to high proviral load and high level of Tax expression. These mechanisms result in high frequency of activated CD4+ T cells. Migration of activated CD4^+^ T cells into different organs such as central nervous system damages surrounding tissue by releasing different cytokines and metalloproteinases (8, 36-37). These immunological abnormalities in T cell function and humoral immunity can also be noticed in SLE (8-9, 11, 15-17). 

This cross-sectional study investigates whether infection with HTLV-I is associated with the development of SLE in a large population of SLE patients in Mashhad or not. Besides a broad literature review compares the results of different surveys carried out on this purpose.

## Materials and Methods

One hundred and thirty women, fulfilling the American College of Rheumatology (ACR) revised criteria (38) for SLE, participated in this study. All participants signed an informed consent form prior to the initiation of the study. The study was approved by Mashhad University of Medical Sciences Ethics Committee.

All patients who were high risk for HTLV-I infection after SLE involvement, including patients with a history of suspicious sexual contact, intravenous drug use, and Medical Centre staff members, were excluded from the study. Consecutive patients without exclusion criteria were recruited from university hospitals, rheumatology clinics, and clinical lupus cohorts between January 2008 and March 2009. Disease activity was determined by the SLE Disease Activity Index (SLEDAI-2k) (39). Demographic and laboratory data were also recorded. Control group was recruited from a cross-sectional study, which was conducted simultaneously in the whole population of Mashhad, to examine the seroprevalence of HTLV-I within the selected households of Mashhad from May to September 2009. Among these participants, 915 individuals were women, who were enrolled as a control group in our study.

*Serological assay and confirmation tests*

Five milliliters of blood were obtained from the patients and the healthy control group and stored at −20°C. Serum samples were screened for HTLV-I antibody by enzyme-linked immunosorbent assay (Diapro, Italy) according to the manufacturer’s instructions. Western Blot (WB) was carried out for all positive ELISA samples for further confirmation of HTLV-I infection in patients and controls. All reactive samples on serologic screening were tested further by Western blot (WB) analysis with the MP Diagnostics HTLV Blot 2.4 (MP Biomedicals Asia Pacific Pte Ltd, Singapore) according to the manufacturer’s instructions. Each anti-HTLV-I positive sample (provided by Abbott Diagnostics) was tested simultaneously with study samples to verify the test results.

*Statistic Analyses*

The statistical analysis was performed using the SPSS 11.5 program (SPSS Inc., Chicago, IL, USA). Values were reported as mean±SD for normally distributed variables and median with interquartile range (IQR) for others. Kolomogrove-Smirnov test was applied for normal variable distribution. Two independent proportion test was used to evaluate statistical differences between the frequency of HTLV-I seropositive results, in patients and control group.

## Results

This cross-sectional study was conducted on 1045 individuals (130 lupus patients and 915 healthy controls). The mean age of the patients was 29.78± 11.37 years. The range of age was 13 to69 years. The mean age of controls was 29.1± 18.5 years with range of 13-55 years. All participants were women. There was no significant difference between the age of the case under study and the control group (*P=*0.9). Two (1.5%) of patients and 23 (2.5%) of controls had HTLV-I infection, which was confirmed by WB (Table 1).

No statistical significant difference was observed between the patients and controls in the number of HTLV-I seropositive samples (*P=*0.49).

The average of the disease duration in the patients was 3 (0.5-5.7) years. The frequency of important lupus manifestations in these patients (according to SLEDAI) are pointed here: renal involvement: 28.3%, seizure: 5.4%, psychosis: 3.3%, severe headache: 5.4%, myositis: 5.4%, arthritis: 33.9%, malar rash/discoid lupus erythematosus (DLE) /alopecia: 42%, serositis: 

21.7%, retinal vasculits: 0.2%, fever: 3%, anemia: 43.4% (hemolytic anemia: 10%), lymphopenia: 40%, leukopenia: 11%, and thrombocytopenia: 14.9%. The mean values of immunologic laboratory parameters in these patients were anti ds-DNA (R): 1.3 (0.5-3) (R is the ratio of the level of anti dsDNA in each patient to the upper limit of the normal range) and C3 (mg/dl): 87 ±46, C4 (mg/dl): 16.6±10 (mg/dl).

**Table 1:** HTLV-I serology in SLE patients compared with control group

Diagnosis	Number tested	HTLV-I antibody positive (percent)	*P*-value
SLE patients	130	2 (1.5%)	0.49
Control group	915	23 (2.5%)	

Clinical features of two HTLV-I seropositive lupus patients were as follows: one patient was 54 years old woman with antinuclear antibody (ANA) (1/180), anti double stranded DNA (ds-DNA) (300 U) and antiphospholipid positive tests in repeated measurements. This patient had lymphopenia (lymphocyte count: 0.75×10^9 ^/L) and seizures due to CNS involvement. The other one, was a 53-year-old patient from Neyshabur (the most endemic city in Khorasan province with regard to HTLV-I infection), with history of HAM/TSP since 4 years before the diagnosis of lupus, which was presented with arthritis, leukopenia (2.3×10^9^/L), lymphopenia (0.6×10^9^/L), positive ANA (1/360) and anti-dsDNA (800 U). The mean age of seropositive patients was 53.5±0.7 years.

As mentioned above, leucopenia and profound lymphopenia were the persistent manifestations in these HTLV-I positive patients. HTLV-I seropositive patients were significantly older than other lupus patients (*P*>0.001, t=-18.9).

## Discussion

Some previous studies have reported the correlations between C-type retrovirus with human autoimmune diseases like SLE (40-41). HTLV-I as a subgroup of C-type retroviruses, plays a pivotal role in various autoimmune diseases (7). Lymphocytes, the main target of HTLV-I usually decline in SLE (18), thus the association between HTLV-I and SLE has been investigated in different endemic areas (9-16, 32). However, the pathogenic role of HTLV-I in systemic lupus erythematosus (SLE) remains controversial.

In the present study, we investigated the association between HTLV-I infection and SLE in the northeast of Iran, Mashhad. This study revealed that HTLV-I infection was not a predisposing factor for SLE in this endemic area, because our SLE patients had the same rate of HTLV-I seroprevalence as the control group.

The results of our study confirm the results of the previous studies in endemic areas of HTLV-I (11, 13-14, 24-27, 30-31). However, few reports have shown that there is a correlation between HTLV-I and some manifestations of SLE (10, 23, 28, 32-33). Some of these studies reported that lupus presentations such as lymphopenia, thrombocytopenia, and nephritis might be associated with HTLV-I infection (32-33). In our study, lymphopenia was seen in both HTLV-I infected patients, but other manifestations of these patients were not different from the features of HTLV-I seronegative patients. However, it is difficult to compare the clinical features between those two groups, due to the small number of HTLV-I seropositive patients. Difference in age of seropositive and seronegative patients in our study was in agreement with Akimoto *et al.* report. The age of HTLV-I-seropositive patients with SLE was significantly higher than that of seronegative patients. Furthermore, the age at onset of SLE in HTLV-I-seropositive cases was also significantly higher than that of seronegative cases, suggesting that HTLV-I may induce a unique autoimmune disease similar to SLE, many years after initial infection (23). The immunological abnormalities such as decreased T cell numbers, impaired T cell function, and existence of lymphocytotoxic antibodies against suppressor T cells have been explained in SLE patients (15-16). However, Bowness, *et al* evaluated the correlation between HTLV-I infection and different autoimmune diseases. They found no antibody against HTLV-I in seropositive patients. In addition, in seropositive HTLV-I group, nobody had positive antinuclear antibodies or antibodies to U1RNP(42). Gourley *et al.* suggested that type Cretroviruses played a role in immune activation in murine lupus (43). Nevertheless, most of the studies could not show any relationship between HTLV-I and lupus (11, 13-14, 24-27, 30-31). The relationship between HTLV-I and SLE in different studies is summarized in Table 2.

**Table 2:** The relationship between HTLV-I and SLE in different studies

The Place of study	Study group(n)	Methods of HTLV-I detection	Number and percent of HTLV-I positivity in patients/ control group	Relationship between HTLV-I & SLE (p-value)	Reference
Japan	SLE (51) , control (37)	ELISA, Gel electrophoresis techniques	1SLE(2%)/ 0	_	Koike *et al *(1985)^24^
Japan/Nagasaki	SLE (10), RA (14), MCTD (4), Behcet D (3), sjögren (4), Dermatomyositis (2)	Indirect IF/ RIA	1SLE patient with RIA technique/ 0	_	Kurata *et al *(1985)^26^
USA/Maryland	SLE (30), control (20)	ELISA, Nucleic acid hybridization techniques	0	_	Boumpas* et al* (1986)^13^
Sweden/Stockholm	SLE (11), RA (27), polymyositis (18), normal (60)	ELISA, Western blot	Non of RA&PM ,1SLE/ 0	_	Lolli *et al* (1987)^25^
Southern Africa/Rankuwa	SLE (12), DLE (34), non lupus patients (34), normal (25)	Indirect IF, WB	12SLE, 1DLE, 1non lupus/ 0	+^*^	Oslen *et al *(1987)^28^
Jamaica	SLE (63), control (12829)	ELISA, p24 protein RIA	4 SLE (6.8%)/	_	Murphy *et al* (1988)^14^
Japan/Kagoshima	Case Report	ELISA	712 control (9.9%)		Ito H *et al *(1990)33
USA/South Carolina	Letter to editor	WB/Radioimmunopercipitation test			Scott *et al *(1990)^32^
USA/Ohio	SLE (94)	WB	11 (12%) reactive, 29 (31%) indeterminate, 54 (57%) uncreative	_	Danao *et al *(1991)^30^
Japan/Kyoto	SLE (40)	WB, PCR	2	_	Higashi *et al *(1992)^31^
USA, Ohio	patients with connective tissue autoimmune disease (115)	WB	0	_	Bailer *et al *(1994) ^7^
Berlin	SLE (24)	ELISA, PCR	0	_	Lipka *et al* (1996)^11^
Sweden	SLE (69)	RIA	58-68 of 69	+(P<0.0005)	Bengtsson *et al * (1996)29
Japan/Hokkaido	Case Report	WB, PCR	14SLE (15.7%)/		Miura *et al* (1999)^10^
Japan/Nagasaki	SLE (89), control (409)	ECLIA/ PCR or Southern blotting analysis	45(11%)	-	Akimoto *et al* (2007)^23^

Abbreviations: ELISA: enzyme linked immunosorbent assay, IF: immunofluorecence, RIA; radioimmunoassay, WB: western blot, PCR: polymerase chain reaction   * ***P*** **-value was not recorded in the article**

The current study was not without limitations, for example, we used ELISA screening assay for all patients and the level of IgG directed against HTLV-I was detected. It has been reported that HTLV-I antibodies of a non-IgG class of immunoglobulin might not been detected during the screening of HTLV-I infection (44). The strength points of this cross-sectional study were carrying out the study in an endemic area in a large population of SLE patients, along with excluding the patients who had risk factors for contamination with HTLV-I after lupus involvement. Besides, case selection for the control group was provided by a systematic epidemiologic method that demonstrated true prevalence of HTLV-I in Mashhad population.

Taken together, the overall outcome of our research is in line with the most of the studies that have shown no association between HTLV-I infection and SLE. Contradictions about the relationship between HTLV-I and SLE occurrence suggest that differences in ethnic background may contribute to this issue. Other conditions such as geographical and environmental factors should be taken into account.

## Conclusion

This cross-sectional case-control study did not find any association between HTLV-I and SLE. With regard to the previous studies, these controversies may stem from differences in ethnic background. Geographical and environmental factors should also be taken into account.
